# T Cell Repertoire Maturation Induced by Persistent and Latent Viral Infection Is Insufficient to Induce Costimulation Blockade Resistant Organ Allograft Rejection in Mice

**DOI:** 10.3389/fimmu.2018.01371

**Published:** 2018-06-15

**Authors:** Jaclyn R. Espinosa, Danny Mou, Bartley W. Adams, Louis R. DiBernardo, Andrea L. MacDonald, MacKenzie McRae, Allison N. Miller, Mingqing Song, Linda L. Stempora, Jun Wang, Neal N. Iwakoshi, Allan D. Kirk

**Affiliations:** ^1^Department of Surgery, Emory University, Atlanta, GA, United States; ^2^Department of Surgery, Duke University, Durham, NC, United States; ^3^Department of Pathology, Duke University, Durham, NC, United States; ^4^Duke University School of Medicine, Durham, NC, United States

**Keywords:** costimulation blockade, T cell memory, viral infections, heterotopic heart transplantation, alloimmunity

## Abstract

CD28:CD80/86 pathway costimulation blockade (CoB) with the CD80/86-specific fusion protein CTLA4-Ig prevents T cell-mediated allograft rejection in mice. However, in humans, transplantation with CoB has been hampered by CoB-resistant rejection (CoBRR). CoBRR has been attributed in part to pathogen-driven T cell repertoire maturation and resultant heterologous alloreactive memory. This has been demonstrated experimentally in mice. However, prior murine models have used viral pathogens, CoB regimens, graft types, and/or antigen systems atypically encountered clinically. We therefore sought to explore whether CoBRR would emerge in a model of virus-induced memory differentiation designed to more closely mimic clinical conditions. Specifically, we examined mouse homologs of clinically prevalent viruses including murine polyomavirus, cytomegalovirus, and gammaherpesvirus 68 in the presence of clinically relevant maintenance CoB regimens using a fully MHC-mismatched, vascularized allograft model. Infected mice developed a significant, sustained increase in effector memory T cells consistent with that seen in humans, but neither developed heterologous alloreactivity nor rejected primarily vascularized heterotopic heart transplants at an increased rate compared with uninfected mice. These results indicate that memory acquisition alone is insufficient to provoke CoBRR and suggest that knowledge of prior latent or persistent viral infection may have limited utility in anticipating heterologous CoB-resistant alloimmunity.

## Introduction

Costimulation blockade (CoB) with the CD80/86-specific fusion protein CTLA4-Ig has long been known to prevent T cell-mediated allograft rejection in mice (1–3). Dramatic and lasting anti-rejection effects have been achieved with short-term CTLA4-Ig treatments, and appropriately spurred substantial translational work to develop CoB-based therapies for use in clinical organ transplantation (4, 5). Indeed, as CoB with CTLA4-Ig, or a higher affinity analog, belatacept, has transitioned into the clinic, patients treated with belatacept have had significantly better long-term graft function and survival compared with patients treated with standard calcineurin inhibitors (6, 7). Despite the long-term benefits of belatacept, pervasive acute CoB-resistant rejection (CoBRR) has hampered its clinical usage. As such, studies into the nature of CoBRR hold importance in realizing the beneficial impact of CoB in human transplantation.

The prevailing mechanism thought to give rise to CoBRR has been T cell repertoire maturation driven by ongoing environmental pathogen exposure, and the attendant decrease in costimulation dependence of increasingly differentiated T cells (8–10). Young, specific pathogen-free laboratory mice are immunologically naïve, particularly when compared with non-human primates and humans presenting for organ transplantation. Humans are exposed to myriad antigens throughout life, and this growing antigen experience results in a phenotypic shift of the lymphocyte repertoire so as to decrease the repertoire’s general dependence on costimulatory pathways, like the CD28:CD80/86 pathway, for activation. Teleologically, this enables cells to more rapidly mount potent recall responses upon subsequent antigen encounters (11–15). Indeed, seminal preclinical studies investigating the relationship of pathogen exposure to CoBRR have clearly shown that CoB-resistant memory can be induced by exposure to some viruses, and in some settings be associated with resultant CoBRR (16–19). Recent clinical data have, however, found no correlation between prior viral infection and acute allograft rejection in patients on conventional immunosuppressive medications (20), and no overt associations between viral exposure and rejection have emerged in early studies involving belatacept (21–24). Thus, the processes that foster costimulation-resistant alloimmunity remain incompletely defined, and their study remains relevant to elucidate the mechanisms of and facilitate means of anticipating patients at risk for, CoBRR.

While heterologous alloimmunity and CoB resistance can clearly be provoked in mice through acute viral exposure, the changes fostered by chronic viral exposure remain undefined, and the degree to which these mechanisms carry over into the clinic has not been established. Specifically, numerous characteristics of prior experiments used to investigate CoBRR in mice have differed markedly from conditions encountered in the clinic. Previous studies have utilized OVA-expressing viruses and OVA-expressing allografts to model direct molecular mimicry (19, 25), rather than the broader spectrums of cross-reactivity that are possible across allogeneic barriers (26–28). Furthermore, the viruses used in many mouse models of memory such as lymphocytic choriomeningitis virus and vaccinia virus are rarely of consequence clinically (29–33), and the viral-specific effects of these pathogens have not been segregated from the more general effects of viral exposure such as bulk T cell repertoire maturation. With respect to immunosuppression, many studies have combined CD28:CD80/86 blockade with CD40:CD154 blockade (1, 2, 4, 34–38), though the latter agent is not used clinically. Furthermore, the type of allograft used in mouse studies has often been secondarily vascularized skin or bone marrow chimeras (16, 18, 34, 37), both of which are convenient and have rapid readout, but differ substantially from vascularized organ allografts. Finally, many mouse studies use a short course of CoB rather than an indefinitely administered maintenance regimen used clinically. While each of these differences are valid experimental techniques that provide unique, well-controlled options for understanding aspects of CoBRR, we sought to design a model to determine whether viral exposure consistent with that known to occur and be diagnostically assessed in humans leads to CoBRR using a CoB regimen used clinically.

We developed a murine model utilizing persistent and chronic viruses that are common and routinely problematic in the transplant population to induce a more terminally differentiated immune profile in mice. Moreover, we employed a primarily vascularized heterotopic heart transplant model, so vasculopathy could be observed and assessed, along with immunosuppressive agents already approved for clinical use, to facilitate translation of experimental findings to the clinic. We find that despite substantial repertoire change following viral exposure, CoBRR is not readily induced by latent or persistent viral infection in the presence of chronic immunosuppression.

## Materials and Methods

### Animals and Viral Infections

C57BL/6 (H-2^b^) and Balb/c (H-2^d^) mice were purchased from the Jackson Laboratory (Bar Harbor, ME, USA). All mice were housed in a specific pathogen-free barrier facility and were 8–12 weeks of age at the beginning of the experiment. C57BL/6 mice were mock infected with sterile PBS, infected with 1 × 10^6^ PFU/mL of polyomavirus (PyV), 1 × 10^5^ PFU/mL of murine cytomegalovirus (mCMV), 1 × 10^5^ PFU/mL of murine gammaherpesvirus 68 (HV68), or all three. PyV was administered *via* footpad injection in 50 μL sterile PBS per foot. mCMV and HV68 were administered *via* intraperitoneal injection in 250 μL of sterile PBS.

**Figure 1:**
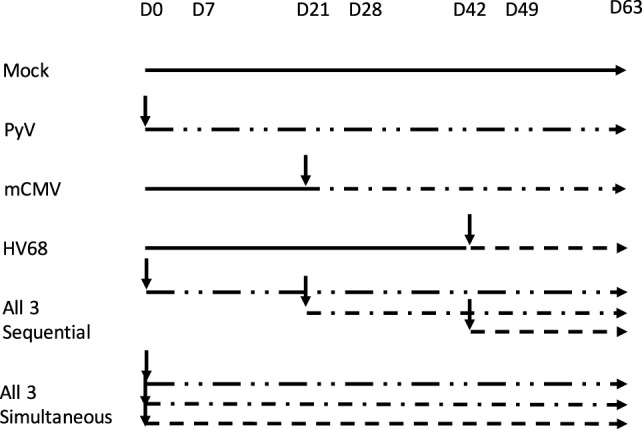
Experimental design for sequential and simultaneous viral infections of C57BL/6 mice. Mice that received single or sequential polyomavirus (PyV) infection were injected on day 0, all other groups received sterile PBS injections. Mice that received single or sequential murine cytomegalovirus (mCMV) infection were injected on day 21; all other groups received sterile PBS injections. Mice that received single or sequential HV68 infection were injected on day 42, all other groups received sterile PBS injections. Mice that received simultaneous infections were injected with PyV, mCMV, and HV68 on day 0, mock-infected mice received sterile PBS injections.

Mice that received all three infections sequentially were bled on day 0, prior to viral exposure, then infected with PyV, and bled at peak (day 7) and memory (day 21) time points to track changes in peripheral blood mononuclear cells (PBMCs). On day 21, mice were then infected with mCMV, and bled on days 28 and 42. On day 42, mice were finally infected with HV68 and bled on days 49 and 63 post-infection. Mice that received single infections were injected on the same day the mice that received all three were infected: PyV on day 0, mCMV on day 21, and HV68 on day 42. Mice that received mock infections or mice that were only infected with one virus were injected with sterile PBS as a control for potential activation due to the injection. Mice that received all three infections simultaneously were bled on day 0, then received either mock or all three infections and subsequently bled on days 7, 14, 21, 42, and 69 post-infection to track changes in immune cell repertoire. Viral infection scheme is depicted in Figure 1.

### Heterotopic Heart Transplantation and Immunosuppression

Balb/c donor hearts were transplanted into the abdomen of C57BL/6 recipients using a modified version of the methods previously described by Corry et al. (39). Briefly, the C57BL/6 recipient mouse was anesthetized with isoflurane. A segment of descending aorta and vena cava below the renal vessels was dissected. The heart was immediately removed from the Balb/c donor and placed in cold University of Wisconsin preservation solution on ice. The Balb/c donor heart was then placed in the abdominal cavity of the recipient, and the donor aorta and pulmonary artery were anastomosed in an end-to-side manner to the recipient abdominal aorta and vena cava using 10-0 monofilament suture. Mice that received chronic CTLA4-Ig were given 250 μg per dose *via* intraperitoneal (I.P.) injection on days 0, 2, 4, 7, and weekly until sacrifice. Mice that received chronic CTLA4-Ig and rapamycin were given 250 μg of CTLA4-Ig per dose on days 0, 2, 4, 7, and weekly and 2 μg of rapamycin every Monday, Wednesday, and Friday *via* I.P. injection until sacrificed. Mice that received short course CTLA4-Ig were given 250 μg per dose *via* I.P. injection on days 0, 2, 4, and 7. Allografts were monitored by palpation, and mice were sacrificed upon rejection (beating cessation) or at the end of the study.

### Flow Cytometry

Cells were isolated from the blood to track serial changes in peripheral blood phenotype, and from the spleen at terminal endpoints. Cells were incubated with a live/dead fixable blue dead cell stain kit (Invitrogen #L34962), then stained with CD3 (BD Biosciences, clone 145-2C11, catalog #551163), CD4 (BD Biosciences, clone RM4-5, catalog #563106), CD8 (BioLegend, clone 53.6-7, catalog #100741 or #100712), CD44 (BD Biosciences, clone IM7, catalog #559250 or #562464), CCR7 (BioLegend, clone 4B12, catalog #120106), CD62L (BioLegend, clone MEL-14, catalog #104406), CTLA4 (BioLegend, clone UC10-4B9, catalog #106306 or 106310), CD28 (BioLegend, clone E18, catalog #122014), PD1 (BioLegend, clone 29F.1A12, catalog #135220), KLRG1 (BD Biosciences, clone 2F1, catalog #562897), CD127 (eBioscience, clone A7R34, catalog #47-1271-82), and CXCR3 (BioLegend, clone CXCR3-173, catalog #126522) for 30 min, then washed and fixed for flow cytometric analysis, or went on to be stained intracellularly. For intracellular cytokine staining, cells were incubated for at least 30 min with fixation/permeabilization buffer, then washed with permeabilization buffer (from eBioscience Factor Staining Buffer Set #00-5523-00) and incubated with interferon gamma (IFNg) (BioLegend, clone XMG1.2, catalog #505808) for 30 min. Cells were then washed with permeabilization buffer and run on a BD LSRFortessa.

Our general gating strategy involved an initial gate on general lymphocytes, then single cells, followed by a live/dead stain to include only live CD3-positive T cells in our analyses. We then gated on CD4 or CD8-positive cells, and finally on different subsets of interest. Memory cells were defined by expression of CD44 and CCR7: naïve (CD44−CCR7+), TCM (CD44+CCR7+), and TEM (CD44+CCR7−), as previously described (40).

### Human Samples

Patients with renal failure were enrolled in an IRB-approved immune monitoring protocol at Emory University (Approval No. IRB00006248). PBMCs were collected from 24 patients prior to transplantation or drug administration and banked for batched analysis. For analysis, aliquots were thawed and interrogated for markers of memory by flow cytometry. Samples were first gated on general lymphocytes, then single cells followed by live CD3+ (BD Biosciences, clone UCHT1, catalog #557943), CD14− (Invitrogen, clone TuK4, catalog #MHCD1430), and CD20− (Invitrogen, clone H147, catalog #MHCD2030) T cells. We then gated on CD4 (BD Biosciences, clone RPA-T4, catalog #560345) or CD8 (eBioscience, clone RPA-T8, catalog #561453) positive populations and finally on memory subsets based on expression of CCR7 (BD Biosciences, clone 3D12, catalog #557648) and CD45RA (Invitrogen, clone MEM-56, catalog #Q10069): naïve (CCR7+CD45RA+), TCM (CCR7+CD45RA−), TEM (CCR7−CD45RA−), and TEMRA (CCR7−CD45RA+).

### Histology

Donor heart allografts were harvested upon sacrifice and placed in 10% buffered formalin for histology. Hematoxylin and eosin staining was performed to visualize allograft damage and cellular infiltrate. Stained slides were de-identified and sent to a cardiothoracic pathologist for blinded assessment of rejection. Allograft rejection was scored using the ISHLT-2004 acute cellular rejection grading scheme as follows: 0R indicates no rejection; 1R indicates mild rejection defined by interstitial and/or perivascular infiltrate with up to one focus of myocyte damage; 2R indicates moderate rejection defined by two or more foci of infiltrate with associated myocyte damage; 3R indicates severe rejection defined by diffuse infiltrate with multifocal myocyte damage ± edema ± hemorrhage ± vasculitis.

### DNA Extraction and Viral PCR

DNA was extracted from donor heart allografts per protocol “Purification of Total DNA from Animal Tissues” with Qiagen kit #69504. PCR was performed to determine whether viral DNA had infiltrated the grafts using TaqMan primers and probes. PyV primer sequences: CGCACATACTGCTGGAAGAAG and TCTTGGTCGCTTTCTGGATAC. PyV probe sequence: 6FAMATCCTTGTGTTGCTGAGCCCGATGAMGBNFQ. mCMV primer sequences: AGGGCTTGGAGAGGACCTACA and GCCCGTCGGCAGTCTAGTC. mCMV probe sequence: 6FAMAGCTAGACGACAGCCAACMGBNFQ. HV68 primer sequences: GGCCGCAGACATTTAATGAC and GCCTCAACTTCTCTGGATATGCC. HV68 probe sequence: 6FAMATTTGGGCGCAATGTGTTGGATGAATAMRA.

### Viral Peptide and Allogeneic Stimulation Assays

Upon sacrifice, splenocytes from experimental C57Bl/6 mice were isolated and stimulated with allogeneic (Balb/c) splenocytes, viral peptides from GenScript USA Inc. for HV68 (p56, sequence: AGPHNDMEI; p79, sequence: TSINFVKI), or viral peptides from JPT Peptide Technologies GmbH for mCMV (M38, sequence: SSPPMFRV; M45, sequence: HGIRMASFI; IE3, sequence: RALEYKNL; M139, sequence: TVYGFCLL). After 1 h of stimulation, Brefeldin A was added to inhibit protein transport from the ER to the Golgi to ensure cytokines being produced would be detected within the cell *via* intracellular cytokine staining. Cells were incubated for a total of 5 h, then washed, and stained for IFNg production as described above.

### Statistical Analysis

Differences in memory cell subsets, expansion of effector memory T cells, KLRG1+ populations and IFNg production between mock-infected and infected mice were calculated using two-way ANOVA. For additional flow cytometry analyses, *p* values were calculated using unpaired *t* tests and one-way ANOVA. Allograft survival data were assessed using the Log-rank (Mantel–Cox) test for statistical significance. For immunohistochemistry, differences in grades of rejection between mock-infected and infected mice were calculated using nonparametric Mann–Whitney test. Statistical methods used for analysis are detailed in each figure legend where *p* values are reported, and *p* values of less than 0.05 were considered statistically significant. Analyses were performed using GraphPad Prism (GraphPad Software Inc., San Diego, CA, USA).

## Results

### Infection With Latent and Persistent Viruses Permanently Alters the Murine Immune Cell Repertoire

**Figure 2:**
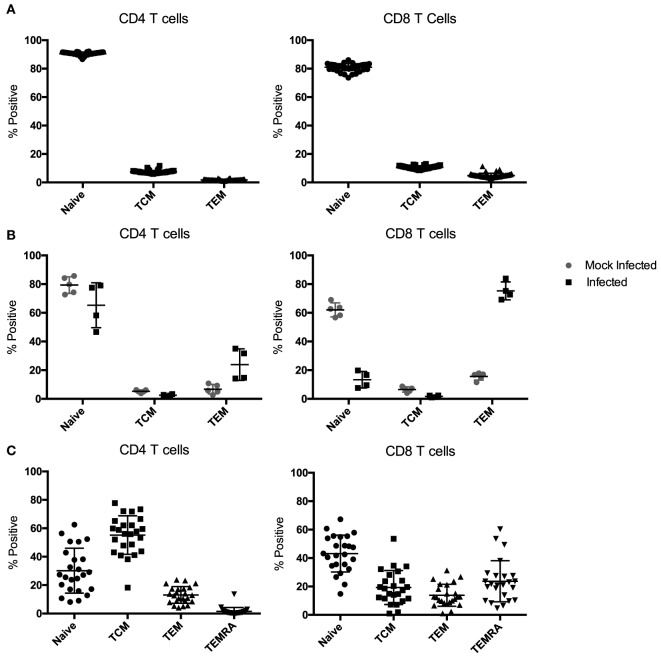
Memory subset distribution of CD4 and CD8 T cells in naïve and infected mice. **(A)** Peripheral blood samples from naive C57BL/6 mice have an almost exclusively naïve immune cell repertoire in both the CD4 and CD8 T cell compartments (*n* = 50). **(B)** Mice infected with polyomavirus (PyV), murine cytomegalovirus (mCMV), and HV68 exhibit a sustained, more diverse immune cell repertoire compared with mock-infected mice up to 70 days post-infection (*n* = 5 per group). **(C)** Memory subset distribution of CD4 and CD8 T cells in adult human subjects is diverse and variable (*n* = 24).

**Figure 3:**
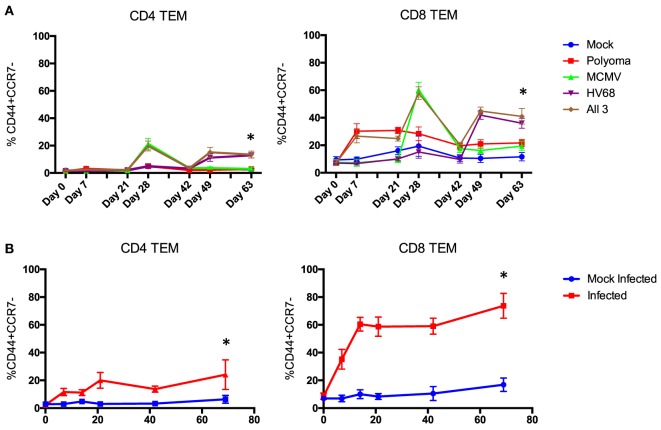
Time course of the change in peripheral blood phenotype in C57BL/6 mice following infection with polyomavirus (PyV), murine cytomegalovirus (mCMV), and HV68. Sequential **(A)** and simultaneous **(B)** infections with multiple viruses result in a significant and sustained increase in TEM cells in both the CD4 and CD8 T cell compartments compared with mice that received mock infections (*n* = 10 per group, *p* < 0.001 calculated using two-way ANOVA).

Most mouse transplant models utilize relatively young, immune-naïve mice, whereas human kidney transplant recipients have considerable immune experience [the median age of transplant recipients exceeds 50 years (41), and even pediatric transplant recipients have experienced substantial repertoire maturation at the time of transplant (42)]. We determined the memory distribution of CD4 and CD8 T cells from 50 C57BL/6 mice maintained in general laboratory conditions. As shown in Figure 2A, all had predominantly naïve repertoires. To assess whether changes in repertoire maturation would lead to meaningful changes in allo-specific reactivity or foster enhanced allograft rejection, we developed a mouse model with a more memory-laden immune cell repertoire. We infected mice with latent and/or persistent virus homologs known to be common in the human population and precarious in the setting of transplantation. We sequentially infected mice with PyV (BK virus homolog), mCMV (CMV homolog), and murine gammaherpesvirus 68 (HV68, EBV homolog). The experimental design is depicted in Figure 1. Each infection resulted in an increase in both CD4 and CD8 TEM cells (*p* < 0.001), with the biggest increase in CD8 TEM in mice that received all three infections (Figure 3A).

Next, we sought to determine whether all three infections could be given on the same day and yield a similar change in CD4 and CD8 TEM while condensing the experimental timeline. Mice were bled on day 0, then received either mock or all three infections and were bled on days 7, 14, 21, 42, and 69 post-infection to track changes in immune cell repertoire. Simultaneous infection with all three viruses resulted in a comparable sustained increase in TEM (*p* < 0.001) in both the CD4 and CD8 T cell compartments compared with mice that received mock infections (Figure 3B), thus establishing a model of murine memory differentiation due to infection with clinically pertinent persistent and latent viruses. The magnitude and nature of the differentiation observed in infected mice (Figure 2B) more closely resembles the diversity observed in human patients (Figure 2C).

### Long-Lived, High Quality Memory Cells Are Induced Following Infection

**Figure 4:**
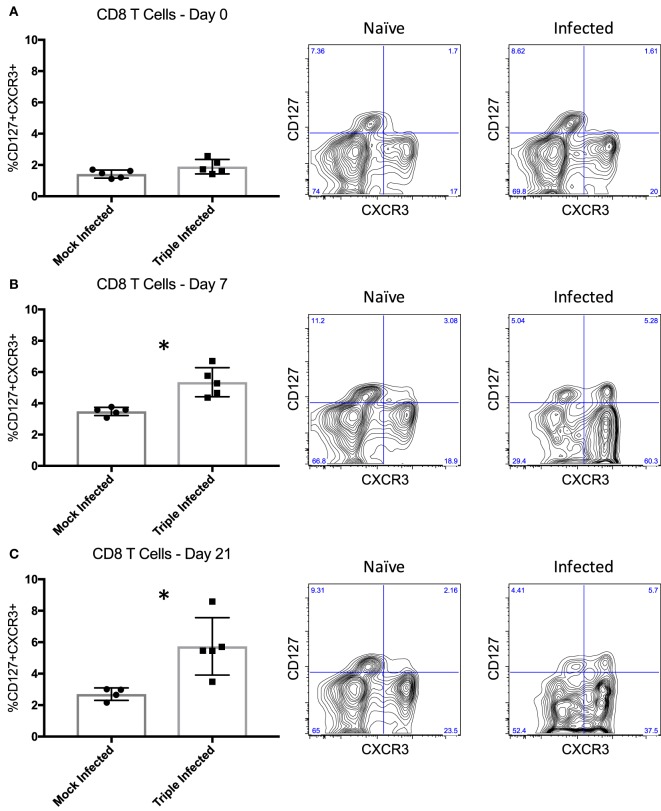
High quality memory cells emerge following infection with polyomavirus, murine cytomegalovirus, and HV68. **(A)** Prior to infection, mice do not have a distinct population of CD127+CXCR3+ CD8 T cells indicative of quality memory (*n* = 5 per group). **(B)** On day 7 post-infection, there is a significant increase in CD127+CXCR3+ CD8 T cells (*p* = 0.0025, calculated using unpaired *t* test). **(C)** The increase of CD127+CXCR3+ cells is sustained through day 21 post-infection (*p* = 0.0146, calculated using unpaired *t* test). Representative flow plots of peripheral blood mononuclear cells from naïve and infected mice are shown to the right of the summary data for each time point.

In addition to memory formation in general, evaluating the quality of those memory T cells may yield greater insight into their ability to elicit recall responses, such that qualitative differences in response to various pathogens may contribute to breadth of heterologous alloreactivity and thus CoBRR. Previous studies have shown that activation phenotype, rather than memory subset, may be a better indicator of the quality, and thus recall efficiency, of memory cells (43). CD8 T cells expressing CXCR3, a chemokine receptor upregulated on activated T cells (44), and CD127, the IL-7 receptor alpha chain expressed on cells known to become long-lived memory cells (45), were identified as being able to mount the strongest recall response to viral infection. Thus, we assessed the expression of CD127 and CXCR3 on PBMCs following infection with PyV, mCMV, and HV68 to determine whether these viruses give rise to CD127+CXCR3+ cells with potent recall responses that may be alloreactive and mediate CoBRR. Prior to infection, mice did not have a distinct population of CD127+CXCR3+ CD8 T cells (Figure 4A). However, following infection with PyV, mCMV, and HV68, there was a significant increase in CD127+CXCR3+ CD8 T cells at the peak (*p* = 0.0025, Figure 4B) and memory (*p* = 0.0146, Figure 4C) time points compared with mice that received mock infections, indicating infection with PyV, mCMV, and HV68 results in the emergence of a long-lived, quality memory cell population. Moreover, all of the CD127+CXCR3+ cells were CD44hi (data not shown), indicating that they were antigen-experienced cells with the capacity to elicit potent recall responses to viral re-stimulation, or allogeneic antigen in the case of heterologous immunity, and did not simply emerge as a result of aging.

### Increase in Terminally Differentiated, but Not Exhausted, T Cells Following Infection

**Figure 5:**
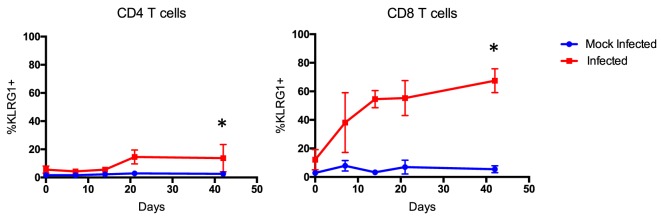
Terminally differentiated cells arise following multiple infections. Expression of KLRG1 was significantly upregulated on CD4 (*p* < 0.0126) and CD8 T cells (*p* < 0.0001, calculated using two-way ANOVA) of mice that received all three infections compared with mice that received mock infections (*n* = 10 per group).

Importantly, expression of other markers of differentiation, like KLRG1, and inhibition, like PD1 and CTLA4, is also imperative to assess and identify antigen-experienced cells that may be implicated in CoBRR (46–50). CTLA4 is particularly of interest in the context of CoB, since CTLA4-Ig inhibits both CD28 costimulation and CTLA4 coinhibition (51). In our murine model of memory formation, the expression of KLRG1 was significantly upregulated on CD4 (*p* = 0.0126) and CD8 (*p* < 0.0001) T cells in mice that received all three infections compared with mock-infected mice (Figure 5). This is consistent with previous studies that have shown KLRG1 upregulation in mice in response to chronic viral antigen exposure (52, 53). Surprisingly, expression of PD1 was largely unchanged, and no long-term difference in expression of CTLA4 was observed on antigen-experienced CD4 or CD8 cells in mice that received all three infections compared with mice that received mock infections (Figure S1 in Supplementary Material). These data, combined with the increase in CD44+CD127+CXCR3+ cells described above, indicate that infection with PyV, mCMV, and HV68 results in a permanent shift in the immune cell repertoire toward a higher quality, unexhausted memory-laden phenotype, primed to elicit recall responses upon re-stimulation.

### Latent and Persistent Viral Infections Do Not Influence Allograft Rejection in the Setting of CoB

**Figure 6:**
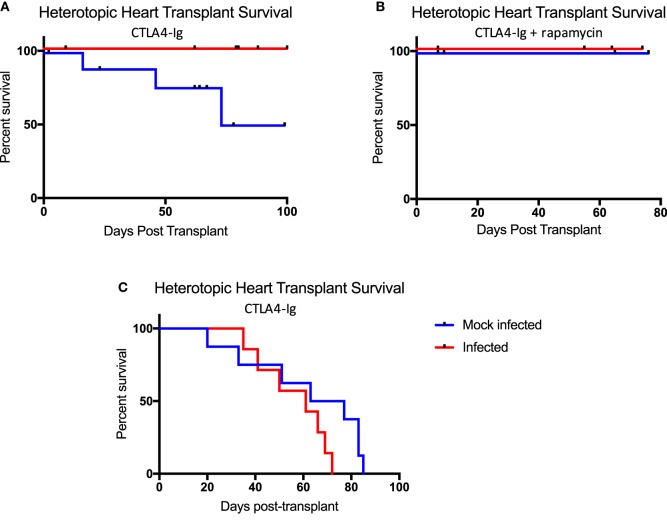
Latent and persistent viral infection does not impact allograft rejection in the setting of costimulation blockade (CoB). **(A)** Mice infected with polyomavirus, murine cytomegalovirus, and HV68 (*n* = 12) under chronic CoB with CTLA4-Ig fail to reject their allograft, while mock-infected mice (*n* = 10) do go on to reject their allograft (*p* = 0.0258). **(B)** No difference in allograft survival was observed between infected (*n* = 4) and mock-infected (*n* = 4) mice under chronic immunosuppression with CTLA4-Ig and rapamycin (*p* > 0.9999). **(C)** When treated with short course CTLA4-Ig, survival curves were not significantly different between infected (*n* = 7) and mock-infected (*n* = 8) mice (*p* = 0.1174). Decrement survival indicates immunosuppressive death with a functioning allograft. Allograft survival data were assessed using the Log-rank (Mantel–Cox) test for statistical significance.

It has been shown using non-MHC antigen-specific, secondarily vascularized skin graft models, that immune responses, sensitivity to immunosuppression therapies, and ultimately allograft outcomes, differ based on the context in which the antigen is delivered (19). These studies have taken advantage of immunosuppressive reagents not yet available clinically. Here, we utilized a primarily vascularized heterotopic heart transplant model and CoB regimens analogous to that used in human kidney transplant patients to explicitly emulate the clinical approach. Belatacept, used clinically, is a CTLA4-Ig fusion protein that inhibits CD28:CD80/86 costimulation to inhibit naïve T cell responses. Rapamycin, which inhibits mTOR signaling, is also used clinically to inhibit memory T cell responses and has been shown to be effective in the setting of kidney transplantation. To test the effectiveness of these immunosuppressive agents in our model, mice were infected with either mock or all three viruses. 40 days post-infection, mice received heterotopic heart transplants and were treated with chronic CTLA4-Ig alone (Figure 6A), or chronic CTLA4-Ig and rapamycin (Figure 6B). While infected mice developed a memory-laden immune profile primed for recall responses prior to transplantation, mice that received all three infections failed to reject their allografts under chronic immunosuppression. Interestingly, some mice that received mock infections did eventually reject their allografts under chronic immunosuppression with CTLA4-Ig, but did not reject their allografts under chronic immunosuppression with CTLA4-Ig and rapamycin.

Though infected mice were unable to reject their allografts under chronic immunosuppression, it was not evident that this was due to the continued presence of CTLA4-Ig, or its ability to induce tolerance. Thus, we repeated our infection protocol followed by heterotopic heart transplantation, but abbreviated our immunosuppression regimen to just a short course of CTLA4-Ig administered in four doses for 1 week post-transplant. Following this regimen, allograft survival in both groups was consistent with previous studies utilizing short course immunosuppression and was not significantly different between the two groups (Figure 6C).

**Figure 7:**
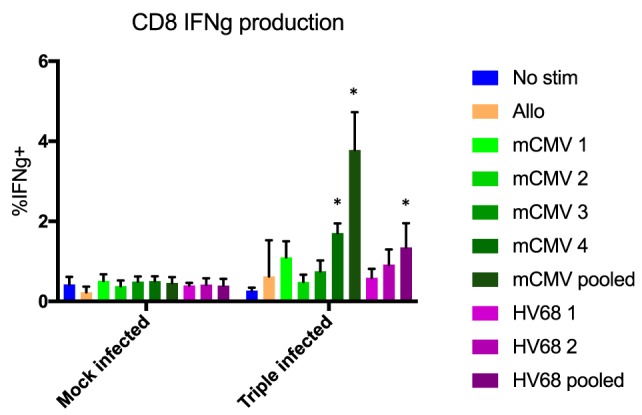
No difference in interferon gamma (IFNg) production was observed in response to any stimulation condition in mock-infected mice. CD44+ CD8 T cells from mice that received all three infections produced IFNg in response to viral peptides, with the highest level of IFNg production in response to pooled peptides. No significant difference in IFNg production between mock- and triple-infected mice in the presence of no stim or allogeneic antigen, but significant increases in triple-infected mice compared with mock-infected mice were observed in the presence of a single or pooled murine cytomegalovirus (mCMV) peptides (*p* < 0.001, calculated using two-way ANOVA), and pooled HV68 peptides (*p* = 0.0013, calculated using two-way ANOVA), *n* = 5 per group.

In addition, to determine whether the lack of difference in rejection rates between groups was due to the cells’ ability to respond to antigen re-stimulation, we performed an *in vitro* activation assay and measured IFNg production. C57BL/6 mice that were either mock-infected or infected with PyV, mCMV, and HV68 were sacrificed 40 days post-infection and splenocytes were co-incubated for 5 h with media alone, allogeneic (Balb/c) splenocytes, or viral peptides to assess IFNg production. We specifically analyzed IFNg production from CD44+ antigen-experienced CD8 T cells, which includes quality memory cells primed for recall responses described above. No difference in IFNg production was observed in response to any stimulation condition in mock-infected mice. However, mice that received all three infections were able to produce IFNg in response to different viral peptides, with the highest level of IFNg production in response to pooled peptides (Figure 7). This is in line with established studies demonstrating the quantity of antigen-specific cells present correlates with the response. Thus, the lack of difference in allograft survival was not due to the inability of memory cells to respond to re-stimulation, but rather the lack of allo-specific memory T cells as a result of viral infection.

### Evidence of Chronic Rejection in Mock-Infected Mice and Mice That Received All Three Infections

**Figure 8:**
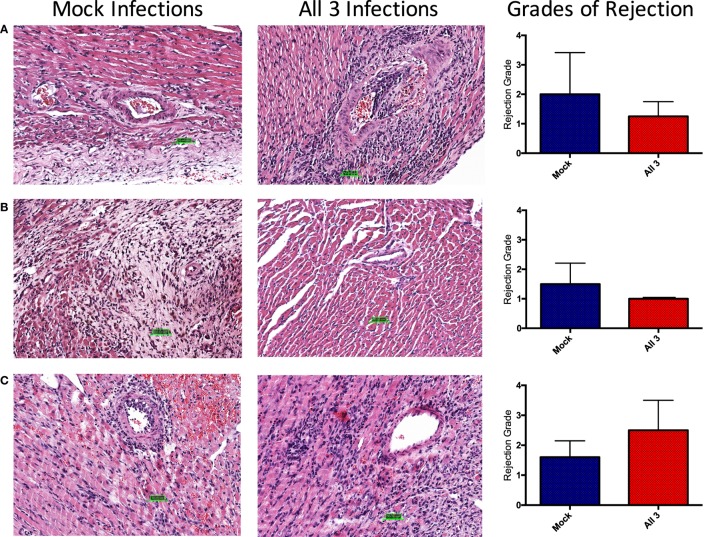
No difference in grades of rejection between mock-infected mice and mice that received all three infections. Donor Balb/c hearts were excised from C57BL/6 recipients at the time of sacrifice. H&E staining was performed, and slides were sent to a cardiac pathologist for blinded analysis. Grades of rejection were scored using the ISHLT-2004 acute cellular rejection grading scheme. A score of 0 indicates no rejection, 1 indicates mild rejection, 2 indicates moderate rejection, and 3 indicates severe rejection. **(A)** Representative histology slides from mock-infected mice with PBS or infected with all three viruses that received fully mismatched heterotopic heart transplants, treated with chronic CTLA4-Ig. Summary data show no significant difference in grades of rejection between groups at day 80 post-transplant (*p* = 0.7333). **(B)** Representative slides from mock-infected or infected mice treated with chronic CTLA4-Ig and rapamycin. Summary data show no significant difference in grades of rejection between groups at day 75 post-transplant (*p* = 3333). **(C)** Representative slides from mock-infected or infected mice treated with CTLA4-Ig on days 0, 2, 4, and 7. Summary data show no significant difference in grades of rejection between groups at day 14 post-transplant (*p* = 0.2381). Statistical analysis of grades of rejection between mock-infected and infected mice was performed using nonparametric Mann–Whitney test.

To assess whether differential rejection could be developing without leading to terminal graft loss, upon sacrifice, donor hearts were excised for immunohistochemistry and viral titers. Remarkably, regardless of infection history, cellular infiltrate was observed in all allografts at the time of sacrifice, though there were no appreciable differences in grades of rejection between the groups (Figure 8). The degree of chronic injury observed in these studies was consistent with historical norms defined in the generation of this model and was not different between infected and mock-infected mice. Interestingly, allografts from mice that received all three infections and chronic immunosuppression with CTLA4-Ig (Figure 8A) or CTLA4-Ig and rapamycin (Figure 8B) had slightly (though non-significantly) lower grades of rejection post-transplant, respectively. A trend toward a higher (but not significant) grade of rejection was seen at day 14 post-transplant after just four doses of CTLA4-Ig in mice that received all three infections compared with mock-infected mice (Figure 8C). The increase in cellular infiltrate at day 14 followed by the decrease at later time points shows that chronic CoB is able to control the rejection such that there was no graft loss.

Furthermore, there was no viral DNA present in the heart allografts at the time of sacrifice, signifying lymphocytes that trafficked into the allograft were unlikely to be responding to viral antigen (data not shown). These data indicate that CoB sufficiently impedes immune responses to allogeneic antigen such that allografts are not rejected as long as CTLA4-Ig is present. However, once CoB is no longer in circulation, it is possible that previously hampered cell responses are able to carry out their effector function and reject existing allografts.

## Discussion

Costimulation blockade with belatacept has been shown to prevent allograft rejection with fewer toxicities than more conventional immunosuppressive regimens. However, a sizable minority of patients experience CoBRR, and this has complicated the generalized use of CoB-based therapies. Numerous studies have been performed in mice to determine the cause of CoBRR, although, to date, all have used models that deviate significantly from conditions likely to be encountered in humans. These differences are important to recognize, not to discount the validity of prior studies, but to better understand the penetrance of the involved mechanisms into clinical practice. As clinical transplantation requires numerous trade-offs between competing mechanisms of rejection, the relative impact of a particular mechanism must be weighed against disease-specific risks, and other concerns known to influence graft outcome. Through these studies, we have established a reliable mouse model of memory differentiation using persistent and latent viral homologs to viruses common in humans: PyV, mCMV, and HV68. Both sequential and simultaneous infection with these viruses resulted in significant and permanent expansion of CD4 and CD8 TEM, to levels consistent with those seen in humans, as well as an increase in KLRG1 expression. Although heterologous alloreactivity with atypical acute infections has been shown to significantly decrease allograft survival in the context of CoB, we have observed no increase in CoBRR in mice infected with PyV, mCMV, and HV68 compared with mock-infected mice under varying chronic immunosuppression regimens.

As with clinical transplantation, numerous variables may influence the aggregate outcomes observed. However, it is notable that no animal in our study experienced early or notably aggressive allograft rejection. Thus, when conditions are specifically set to mimic clinical transplantation, we have been unable to demonstrate a meaningful impact of general repertoire differentiation in the pace or character of CoB-resistant allograft rejection. These data, combined with emerging data in non-human primates and humans (54, 55), indicate that general repertoire maturation like that driven by latent herpesviruses, is unlikely to present an insurmountable barrier to the use of CD28:CD80/86 CoB.

These data make clear that the emergence of an increasingly differentiated repertoire is not, in and of itself, synonymous with development of a CoB-resistant repertoire. Although it has been considered *a priori* that the changes in TCR threshold and increased adhesion and avidity that occur with progressive differentiation could pull clones with marginal alloreactivity into a productive allo-specific response, there has not previously been an explicit test of this hypothesis. The results reported herein suggest that meaningful heterologous alloimmunity may be more dependent on direct, high-affinity molecular mimicry, than on broad changes in trafficking or adhesion induced during repertoire maturation. Indeed, if memory cells generated from a viral infection do cross-react with allogeneic antigen, those cells are capable of inducing CoBRR. This has been clearly demonstrated in experiments utilizing viruses tagged with OVA followed by a transplant with an OVA-expressing graft, which further support the necessity of antigen specificity (56). Thus, antigen specificity rather than broad memory formation is likely critical for CoBRR.

It is important to point out that most prior studies of CoBRR in mice have been models of tolerance, not immunosuppression, in that therapeutic immunosuppression has been withdrawn and animals observed for subsequent rejection. Thus, the rejection observed is not necessarily resistant to CoB, but rather, a breakdown in the stability of tolerance established by CoB. Though interesting, it may not foreshadow consequences relevant to individuals continuously receiving therapeutic immunosuppression. That said, in our short course studies, no substantial difference was observed between infected and mock-infected animals.

These data are consistent with recent data in non-human primates indicating that continuous belatacept and rapamycin effectively prevents CoBRR (54, 57). It also is consistent with clinical studies showing that humans can be controlled on belatacept and rapamycin despite routine viral exposures (23). These data are also consistent with observational human studies that fail to demonstrate any increased risk of allograft rejection conferred by prior latent herpesvirus exposure (20–22) and indicate that repertoire maturation alone is insufficient to provoke a capacity for CoBRR under ongoing therapy.

Specific effector populations that are truly resistant to CoB may emerge prior to transplantation and may play a role in CoBRR, much like we have observed in kidney transplant recipients (24). However, even in the setting of complete MHC mismatch, as utilized in this study, where the stochastic potential for heterologous allo-cross reactivity is maximized, broad changes in repertoire maturity do not pull sufficient numbers of CoB-resistant and allo-cross reactive cells into a graft-specific response. Thus, the emergence of a memory T cell population is not in and of itself enough to increase CoBRR, and a pro-inflammatory by-stander effect is unlikely to be a major driver of CoBRR. Viral infections lead to a more terminally differentiated immune cell repertoire, but the viruses used herein did not give rise to T cell clones that were both costimulation-independent *and* alloresponsive in the context of our Balb/c and C57Bl6 MHC-mismatched model.

Development of murine models that meticulously reflect the patient population and build on established mechanistic studies is imperative, not only to determine which cells mediate CoBRR but also to understand their clinical mitigation and anticipation. Here, we have shown that memory cells derived from persistent and latent viral infections do not impact allograft survival in the presence of CoB. Further studies are needed to define the differential impact of acute and latent infections and the emergence of alloreactive memory cells, as well as the distinction between tolerance established by CoB and the development of cells that are truly resistant.

## Ethics Statement

For animal subjects, this study was carried out in accordance with the recommendations of Duke Institutional Animal Care and Use Committee under approved protocol A171-17-07. For human subjects, this study was carried out in accordance with the recommendations of Emory University Institutional Review Board under approved protocol IRB00006248. All subjects gave written informed consent in accordance with the Declaration of Helsinki.

## Author Contributions

JE designed, planned, and carried out the experiments, collected experimental data, performed data analysis, drafted the manuscript, and designed the figures. DM contributed to experimental design, carried out experiments, collected experimental data, and performed data analysis. BA optimized viral titer assays and collected experimental data. LD analyzed and interpreted histology. ALM, MM, and ANM collected, processed, and assembled experimental data. MS processed tissue samples for histology. LS contributed to flow panel design, optimization, and performed data analysis. JW performed heterotopic heart transplants. NI contributed to experimental design, carried out experiments, collected experimental data, and provided critical feedback for data analysis and manuscript preparation. AK conceived and directed the project, provided critical feedback for experimental design and data analysis, interpreted data, and finalized the manuscript.

## Conflict of Interest Statement

The authors declare that the research was conducted in the absence of any commercial or financial relationships that could be construed as a potential conflict of interest.
