# Supplementary figures and images for: T Cell Repertoire Maturation Induced by Persistent and Latent Viral Infection Is Insufficient to Induce Costimulation Blockade Resistant Organ Allograft Rejection in Mice
Source: Front Immunol. 2018 Jun 15;9:1371. doi: 10.3389/fimmu.2018.01371 (PMC6013589; doi:10.3389/fimmu.2018.01371)

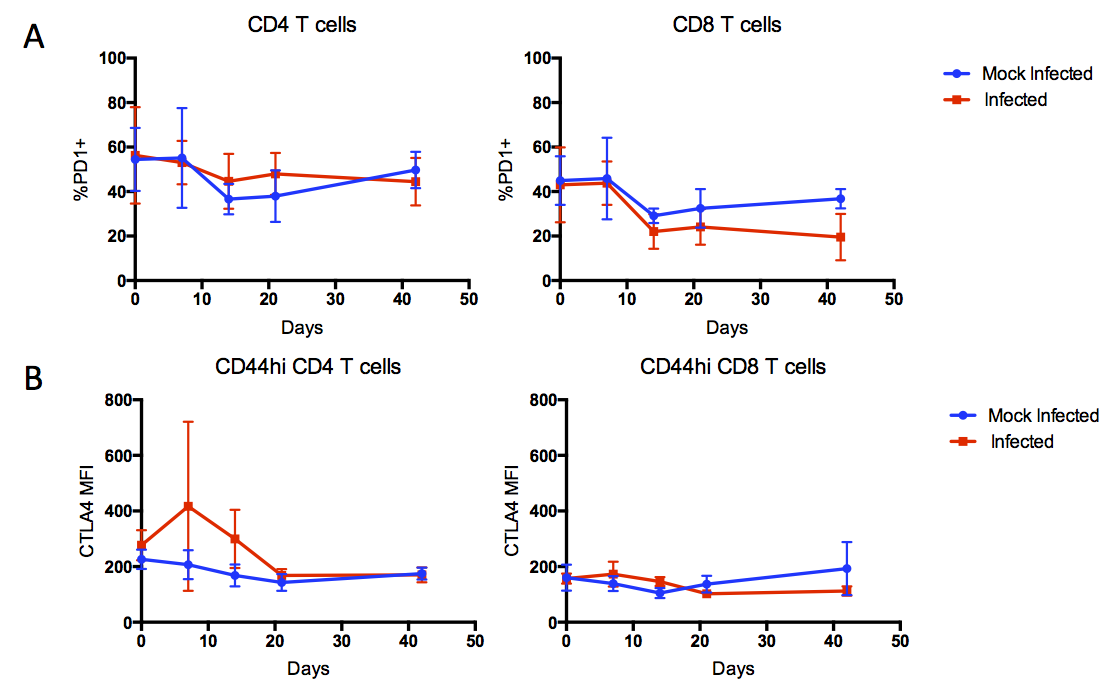

Supplement: Supplementary file 1 [file image_1.tiff]
